# The Games Infants Play: Social Games During Early Mother–Infant Interactions and Their Relationship With Oxytocin

**DOI:** 10.3389/fpsyg.2018.01041

**Published:** 2018-06-25

**Authors:** Gabriela Markova

**Affiliations:** Department of Applied Psychology: Health, Development, Enhancement and Intervention, Faculty of Psychology, University of Vienna, Vienna, Austria

**Keywords:** social play, game routines, mother–infant interactions, oxytocin, engagement

## Abstract

The present study examined early social game routines during natural face-to-face mother–infant interactions and their relationship with oxytocin. Forty-three mother–infant dyads were observed, when infants were 4 months old, during a procedure involving a baseline and a natural interaction, where mothers were instructed to interact with their infants as they would at home. During this procedure four saliva samples from mothers and infants were collected to determine levels of oxytocin at different time points. Social game routines and infant social engagement (gaze, positive, and negative affect) were coded during the natural interaction. Social games were observed in 76.7% of the mother–infant dyads, and 46 different types of games were identified. Mothers initiated games to re-engage infants significantly more often than when infants were already engaged with them. During the games, infants showed more positive affect and less negative affect in comparison to the rest of the interaction. Finally, maternal increase in oxytocin from before to after the natural interaction was positively correlated with game rate and time spent in games, while infant increase in oxytocin from before to after the natural interaction was inversely related to game rate. These results indicate that social games are an inherent part of early mother–infant interactions, and their occurrence is associated with oxytocin of both infants and mothers.

## Introduction

Social play consists of social activities with the goal “to have fun, to interest and be with one another” (Stern, 1977, p. 71). Infants and their caregivers begin to co-construct vocal, gestural, and also multimodal social game routines, such as peek-a-boo, throughout the 1st year of life (e.g., Bruner and Sherwood, 1976; Gustafson et al., 1979; Fantasia et al., 2014b). Recent research suggests that already 3-month-old infants actively participate in such game routines and recognize when their structure is violated (Fantasia et al., 2014b). Despite this evidence, we know little about the contexts and formats of early social game routines and their underlying mechanisms. For example, oxytocin plays a vital role in human social behavior, and seems particularly influential during social interactions between infants and their caregivers. Consequently, the goal of the present study was to investigate naturally occurring social game routines during early face-to-face mother–infant interactions and their relationship with oxytocin.

Early social interactions between infants and their caregivers are characterized by a face-to-face context, close physical contact and a turn-taking structure, where cycles of mutual attention between mothers and infants (engagement) and cycles of non-attention (disengagement) alternate (e.g., Brazelton et al., 1974; Field, 1978; Stern, 1985; Trevarthen, 1993; Papoušek and Papoušek, 1995). Research suggests that these pre-verbal communicative exchanges between adults and infants take place across different modalities, such as through vocalizations, facial expressions, gazes, touch or gestures (e.g., Condon and Sander, 1974; Murray and Trevarthen, 1986; Kobayashi and Kohshima, 2001; Schore, 2001). Early interactions are often characterized as a dialog or a mutual, bidirectional process (Tronick, 1989), in which both partners modulate the timing, the form and the intensity of interaction and their own emotional expression to achieve complementary interactive exchanges (Ammaniti and Trentini, 2009). Thus, the purpose of early engagements is to share meaning, particularly affect with another person (Stern, 1977; Trevarthen and Aitken, 2001; Markova and Legerstee, 2006; Legerstee et al., 2007). Interestingly, these early affective communicative interactions have been described as playful, because their sole purpose is to share experiences with another person (Stern, 1977). Consequently, early social interactions seem to be the optimal context for social play to develop. While some forms of interactions between infants and adults can be clearly characterized as play (e.g., tickling, blowing raspberries), the distinction between what constitutes social interaction as compared to social play is difficult to make (see e.g., Burghardt, 2011). Investigating early social game routines may be one way to distinguish between the two largely overlapping constructs.

Social game routines have a clear recurring structure (Stern, 1977; Crawley et al., 1978; Gustafson et al., 1979; Crawley and Sherrod, 1984; Tamis-LeMonda et al., 2002; Fantasia et al., 2014b), which allows infants to follow elementary rules in their social interactions (Ross and Kay, 1980). Social game routines also follow explicit rules and sequences (Ratner and Bruner, 1978; Ross and Kay, 1980) that are observable at the vocal as well as the motor level of the game. Such games usually include simple rhymes or songs that are associated with motor movements to achieve a coordination of behavior and vocal expressions (Crawley et al., 1978). This visual language (see Hay et al., 1979; Goldman and Ross, 1978) is depicted in **Table 1** for the three most common game routines used by mothers in the sample examined in the current study. Thus, social game routines are characterized by a multimodality that includes a vocal-kinetic format in the form of a rhyme or song, and hand gestures or physical manipulation of the child’s body corresponding to the context of the given rhyme or song (Crawley et al., 1978; Fantasia et al., 2014b).

**Table 1:** Multimodal sequence for the three most common social game routines in the present study.

Steps	Song lyrics	Gestures
**Paci, paci, pacičky**		
1	Paci, paci, pacičky, to jsou moje ručičky [*Clap clap, clap, these are my hands*].	Hold infant’s hands and clap them together.
2	Ťapi, t’api, t’apičky, to jsou moje nožičky [*Stomp, stomp, stomp, these are my feet*].	Hold both infant’s feet and tap them together.
3	Ručky aby dělaly, nožky aby běhaly [*Hands are for doing, feet are for running*],	Move infant’s hands, and then move his/her feet.
4	očka aby viděla, ouška aby slyšela [*eyes are for seeing, ears are for hearing*].	Lightly tap infant’s eyes, and then lightly pull his/her ears.
5.	Pusinka je na papání a nosánek na čmuchání [*The mouth is for eating and the nose is for smelling*].	Lightly tap infant’s mouth, and then lightly tap his/her nose.
**Vařila myšička kašičku**		
1	Vařila myšička kašičku, na zeleném rendlíčku [*Mother mouse cooked porridge in a little green pan*].	Draw a circle in the palm or on the belly of the infant.
2	Tomu dala, tomu víc, tomu málo, tomu nic [*She gave porridge to this baby mouse, little more to this baby mouse, little less to this baby mouse, and nothing to this baby mouse*].	Beginning with the thumb, lightly tap each finger on the infant’s hand.
3	A ten maličký utíkal do komůrečky na homolečky [*And the smallest baby mouse ran to the pantry and found some sugar cones*],	Tap the little finger and then run fingers along the infant’s body toward the mouth or the armpit.
4	A tam se napapal [*and ate as much as he could*].	Put finger into the infant’s mouth, or lightly tickle the infant.
**Kovej, kovej, kováříčku**		
1	Kovej, kovej, kováříčku, okovej mi mou nožičku [*Smite, smite, smith, put a horseshoe on my foot*],	Gently ‘hammer’ at the bottom of the infant’s foot, as if putting a horseshoe on a horse.
2	okovej mi obě, zaplatím já tobě [*put horseshoes on both my feet, and I’ll pay you well*]:	Repeat the action on the other foot.
3	na svatého Víta dám ti pytel žita [*on St. Vitus’ day I’ll give you a sack of rye*,], na svatého Vavřince dám ti pytel pšenice [*on St. Lawrence’s day I’ll give you a sack of wheat*], na svatého Martina dám ti pytel ječmena [*on St. Martin’s day I’ll give you a sack of barley*], a na svatou Barboru dám ti pytel bramborů [*and on St. Barbara’s day I’ll give you a sack of potatoes*].	Symbolically put the different cereals in the hands of the infant.

Various authors have argued that infants are initially passive during a playful interaction, which is initiated by the adult (e.g., Bruner and Sherwood, 1976; Crawley et al., 1978; Ratner and Bruner, 1978; Gustafson et al., 1979; Bruner, 1983; Hodapp et al., 1984; Ross and Lollis, 1987). In the second half of the 1st year infants then assume a more active role in the game that is associated with their growing tendency toward structured games as well as motor capabilities, which allow them to contribute to the games more actively (Bruner and Sherwood, 1976; Crawley et al., 1978; Ratner and Bruner, 1978; Ross and Kay, 1980; Crawley and Sherrod, 1984). In contrast, recent research investigating routine activities suggests that infants actively participate in and contribute to daily routines from early on in life (e.g., Nomikou and Rohlfing, 2011; Rączaszek-Leonardi et al., 2013; Reddy et al., 2013). Routines, such as picking up or changing a diaper, enable infants to recognize their structure and, consequently, built expectations about and thus anticipate others’ behaviors (Reddy et al., 2013). By participating in these shared and meaningful social routines, infants practice their ability to make sense of and coordinate with the others’ actions. In this context, Fantasia et al. (2014b) examined infants’ sensitivity to violations of social game routines. The authors found that structured game routines take place already at 3 months of age and infants are sensitive to modifications of these multimodal routines, such as when mothers leave out the rhyme or gestures of a particular game (Fantasia et al., 2014b). Specifically, infants reduced their body movements and positive vocalizations, and avoided their mothers’ gazes when a game structure was violated (Fantasia et al., 2014b). These findings suggest that already 3-month-old infants have expectations of the structure of early social game routines and recognize when this structure is modified. Unlike free unstructured play, game routines include fixed action patterns, which make them highly predictable. This feature makes it then very easy for even very young infants to actively participate in the games (Fantasia et al., 2014b). Understanding the structure of play as a sequence of tasks, based on the practical, procedural understanding of the routine (Lerner et al., 2011), and not as a necessity for the presence of cognitive representation of the game (Tomasello, 2009), enables infants to be fully capable partners in this shared activity.

Research reviewed thus far suggests that participation in social game routines is cooperative. Interestingly, there is evidence showing that oxytocin (OT) underlies cooperation in adult populations (see Bartz et al., 2011, for review), and this neuropeptide also seems particularly influential during early social interactions in that it enhances social competence of both infants and adults (e.g., Feldman et al., 2007; Levine et al., 2007). For example, an abundance of research has shown that OT promotes parental caregiving behaviors (e.g., Feldman et al., 2010a; Gordon et al., 2010a,b,c; Naber et al., 2010, 2013), and also infant social engagement (Feldman et al., 2010b; Weisman et al., 2012). Most importantly, it is particularly the match between infants’ and parental behaviors that seems to be reflected in the workings of the OT system (Feldman, 2006, 2007; Feldman et al., 2010a, 2011; Gordon et al., 2010b,c). Playing social games involves coordinated activities resulting from a continued attempt to construct and maintain a shared purpose (Tuomela, 2000). Thus, there may be interesting links between playing early social game routines and OT.

In the present study, we set out to examine (1) the occurrence of early social game routines during natural face-to-face mother–infant interactions, (2) infant engagement in these game routines, and (3) their relationship with salivary OT of both mothers and infants. Consequently, we have observed mothers and their 4-month-old infants during a procedure involving a baseline and a natural interaction, during which saliva samples were collected from both mothers and infants to assess OT. During the natural interaction, we have observed naturally occurring social game routines as well as infant social engagement throughout the games and the rest of the interaction. We expected that dyads will spend a considerable amount of time in social game routines and infants will enjoy these game routines more than the rest of the interaction. We also hypothesized that there will be associations between maternal and infant participation in the game routines and their levels of OT.

## Materials and Methods

### Participants

Overall, 43 mothers and their infants (24 girls) participated in the present study. Mothers were recruited in prenatal childbirth classes and in mother–infant activity classes. Their visit to the laboratory was arranged when infants were 4 months (*M* = 139.43 days, *SD* = 19.415 days). All infants were born healthy (5 min Apgar ratings 6–10) and at term, with a gestation period of at least 36 weeks. The majority of infants (93.3%) had no siblings. Mothers were 31.60 years old at infants’ birth (*SD* = 3.578 years) and had on average 5.08 years of higher education (*SD* = 2.853 years). The majority of mothers were primiparous (90.7%) and were breastfeeding their infants (86.7%). All dyads were of European Caucasian origin (Czech) and came from middle to upper class families based on parental education. Mothers and infants received a small gift for participating.

### Procedure and Materials

The Institutional Ethics Committee approved the study. In line with previous research, visits at the laboratory were scheduled between 1 and 4 pm (see Feldman et al., 2010a, 2011). Mothers were asked to come at least 30 min after breastfeeding and to refrain from eating or drinking (other than water) at least 1 h before testing. The mean time difference between last feeding and first saliva collection was *M* = 90 min (*SD* = 34.41, *range* = 15–193 min).

Initially, mothers were informed about the experimental procedure and saliva extraction, after which they signed an informed consent. Mothers were then instructed to rinse their mouth with water to remove food residue and the first salivary sample was collected from mothers and infants. Infants were seated in an infant-seat lying on a table (95 cm × 65 cm × 50 cm) and mothers sat facing the infant (approximate eye level between mothers and infants was 30 cm). The experimenter was present in the room, out of sight from mothers and infants, and did not communicate with infants or mothers during the procedure (except when explaining the procedure). Interactions were filmed using two digital cameras.

There were two main parts of the procedure that were analyzed for the purpose of the present study: a condition without communication (i.e., baseline) and a natural interaction. There was a third condition following the natural interaction, a modified interaction, in which mothers were instructed to change their interaction style (e.g., use adult directed speech). The behavioral analysis of this condition was not part of the present investigation. The three conditions were presented in a fixed order, each lasting approximately 10 min. Consequently, the duration of the procedure ranged between 30 and 40 min. To obtain a baseline where no interaction between mothers and infants takes place, mothers were asked to fill out various questionnaires and to refrain from communicating with infants, while infants watched a Baby Einstein^®^ DVD designed for children from 3 months. During the natural interaction, mothers were instructed to interact with their infants as they usually would at home, without toys. No specific instructions were given pertaining to playing in general, nor playing structured game routines in particular.

#### Saliva Samples

**FIGURE 1:**
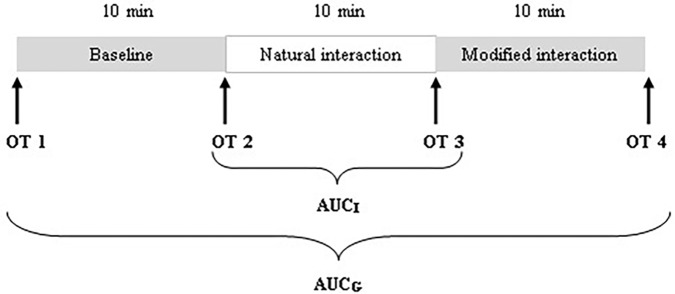
Schematic diagram of the procedure and saliva collection schedule.

During the visit, a total of four saliva samples from each mother and infant were collected using oral swabs to determine the concentration of OT: OT1 was collected after mothers and infants came to the laboratory and were informed about the experimental procedure; OT2 was collected after the baseline; OT3 was collected after the natural interaction; and OT4 was collected after the modified interaction (see **Figure 1**). Mothers were instructed to keep swabs (Salimetrics Oral Swab) under their tongue for 2 min. A research assistant collected saliva samples from the infants (Salimetrics Infant’s Swab). The swabs were put into collection tubes immediately after collection and kept on ice in a thermocol ice box during the whole procedure. After the procedure, collection tubes were frozen and stored at −20°C.

### Measures

All sessions were videotaped and behaviors during the natural interaction were coded from the videos at a later point in time. Behaviors were coded separately and at different times. To determine inter-rater reliability, one rater coded all data and a second rater independently coded 30% of randomly selected data. There was high inter-rater reliability, computed as intra-class correlations, for all behavioral measures (see **Table 2**).

**Table 2:** Inter-rater reliability computed as intra-class correlations (ICC) for all behavioral measures.

Behavioral measures	ICC
Social game routines	0.999
Gazes at mother	0.980
Gazes away	0.994
Positive facial expressions	0.935
Negative facial expressions	0.960
Positive vocalizations	0.944
Negative vocalizations	0.908

#### Social Game Routines

A social game routine was defined as an infant-directed activity. Because early game routines usually contain nursery rhymes in combination with gestures that are dependent on the rhymes’ context, we were particularly looking for games complying with this vocal-kinetic format. Social game routines were coded when: (a) they were recurring and universal across and within dyads; (b) they were individually varied (e.g., gestures/rhymes varied across and/or within dyads), but adhered to the vocal-kinetic format; or (c) their structure was individual (e.g., song that usually does not go along with gestures, but individually performed gestures matched the song’s context), but they adhered to the vocal-kinetic format. Coding of a social game routine commenced when every element (i.e., verbal and non-verbal expressions) of a particular game was present and a game structure was clearly recognizable. Coding was discontinued when the game activity was interrupted or the game was completed. Games that violated the vocal-kinetic format (e.g., mother sang without corresponding gestures), or lacked a recognizable and universal structure (e.g., unstructured play) were not coded. The onset and offset of each game was noted, and the following variables were then computed: (1) game rate was defined as the total number of games, adjusted to the individual duration of each interaction; (2) relative duration of a game was defined as the duration of a game (in seconds) adjusted to the total number of games played; and (3) percentage of time spent in games was defined as the total duration of time spent in games, adjusted to the individual duration of each interaction.

#### Infant Social Engagement

Infant gazes (i.e., gazes at mother, gazes away), facial expressions (i.e., positive, negative), and vocalizations (i.e., positive, negative) were coded second-by-second. The durations of behaviors were adjusted according to the duration of each individual interaction (for similar coding see Peláez-Nogueras et al., 1996; Markova and Legerstee, 2006; Legerstee and Markova, 2007). We coded gazes at mother as infant gazes at their mother’s face, and gazes away as infant gazes away from their mother’s face at something else in their surroundings. To be coded, infant gazes had to last a minimum of 1 s. Positive facial expressions were defined as smiles with the mouth (open or closed) turned upward. Negative facial expressions were coded when infants showed negative emotions like distress, fretting, anger, or discontentment with mouth curled or grimacing. Vocalizations were coded when a discrete sound occurred within one respiration cycle. Two separate sounds were coded if the sound was segmented by a 1 s silence. Vegetative sounds, such as wheezes, sneezes, cough, hiccups, and effort sounds, such as grunting and panting, were excluded. Infant positive vocalizations were produced with a composed facial expression and defined as sounds containing varied pitch contours, produced relaxed and syllable-like, often called babbling, and containing oral resonance. Infant negative vocalizations were produced with an agitated facial expression and defined as vocal sounds that were produced somewhat forced or with effort and were often series of vowel-like sounds, somewhat nasal with uniform pitch, such as whimpers, fusses, cry sounds, and wails. Consistent with previous research (Peláez-Nogueras et al., 1996; Legerstee and Markova, 2007) we computed composite scores for positive and negative affect. Positive affect included positive facial expressions and vocalizations, negative affect included negative facial expression and vocalization. Infant gazes were considered as a separate category.

#### Oxytocin Analysis

The present study used a similar method as previous research validating measurement of OT in saliva (for exact methodology see Carter et al., 2007). However, because measurement of OT in saliva was a relatively new approach without any standardized protocol, a pre-experiment was conducted. The results from the pre-experiment showed that all test samples (5 adults and 5 infants) had sufficient volume concentrations (at least 1 mL) and were above the limit of detection of the assay. Thus, unlike in previous research (Carter et al., 2007; Feldman et al., 2010a,b, 2011), it was not deemed necessary to concentrate the samples before assay. We used a commercially available kit (Oxytocin EIA kit, ADI-901-153, Enzo Life Science) to determine the concentration of OT. The limit for detection of the assay was 11.7 pg/mL. Saliva was recovered from the swabs by centrifugation (2500 ×*g* for 10 min at 4°C). Samples were measured directly without any further modification, and the assay procedure was performed meticulously following the kit’s instructions. All test samples were run in duplicates and a separate standard curve was constructed for each plate. After the first part of the assay procedure, the plate with reagents was incubated overnight at 4°C. On the following day the plate was incubated at room temperature for 1 h. The reaction was then stopped and the optical density of the samples was immediately read on a microplate reader at 405 nm. The concentrations (in pg/mL) of OT were calculated from the relevant standard curve using Softmax Pro 5.2. Each standard curve was checked for quality control parameters as stated in the instructions. The intra-assay coefficient of variability was 13.28%.

## Results

Prior to analyses, all data were screened for deviations from a normal distribution and univariate outliers (*z* > ±3). Outliers were assigned a new score one unit higher/lower than the next highest/lowest score in the distribution (Tabachnick and Fidell, 2001). Because most of the data was not distributed normally, non-parametric tests were used. Data were also screened for possible confounding variables (maternal age, education, breastfeeding, primiparity, symptoms of depression, time difference between last feeding and first saliva extraction, infants’ age and gender), and these were not found associated with any of the behavioral nor OT variables.

Descriptive statistics for behavioral measures are shown in **Table 3**. Gazes at mother were perfectly negatively correlated with gazes away, as was to be expected. Infant gazes at mother were significantly correlated with positive affect displays, *r*(43) = 0.595, *p* < 0.001 (the same negative correlation was found between gazes away and positive affect), and positive affect was negatively correlated with negative affect, *r*(43) = −0.372, *p* = 0.014.

**Table 3:** Descriptive statistics for behavioral measures of the present study.

	*M*	*SD*	Range
Game rate^a^	3.606	3.441	0–14.52
Relative duration of games^b^	10.52 s	8.791 s	0–28 s
Time spent playing games^c^	11.93%	12.60%	0–42.51%
Infant gazes at mothers^c^	39.651%	23.506%	0–86%
Infant gazes away^c^	59.963%	25.527%	0–99%
Infant positive affect^c^	31.137%	22.228%	0–96.10%
Infant negative affect^c^	29.205%	21.526%	0–86.56%

*^a^Rate = total frequency of games, adjusted to the individual duration of each interaction. ^b^Relative duration = duration of a game (in seconds), adjusted to the total number of games played. ^c^Proportional duration = total duration of a particular behavior, adjusted to the individual duration of each interaction.*

### Occurrence of Social Game Routines

Social games were observed in 76.7% of the mother–infant dyads. A comparison between playing and non-playing dyads revealed significantly higher years of maternal education for playing (*M* = 5.52, *SD* = 2.62) than for non-playing mothers (*M* = 3.70, *SD* = 2.16; *z* = 2.210, *p* = 0.031). Comparisons on the study’s main variables or other background variables were non-significant. Mothers and infants spent on average 12% of their interaction time playing games, and games lasted on average 11 s. The first game occurred on average 1.13 min after the onset of the interaction (*SD* = 1.23 min). Overall, 46 different game routines were identified (see Supplementary Table S1 in supplementary materials for a list of all observed games and their frequency of occurrence), and 37% of these games were played by at least two different dyads.

The three most common game routines were *Paci, paci, pacičky* (39.53%), *Vařila myšička kašičku* (30.23%), and *Kovej, kovej, kováříčku* (20.93%; see **Table 1** for the detailed sequences of these game routines). During *Paci, paci, pacičky* the whole body of the infant is used. The game begins by the mother clapping the hands of her infant and indicating how hands can be used. Then the mother moves to stamping the infant’s feet, lightly pulling his/her ears or tapping his/her mouth with her finger, each time indicating what these body parts are used for. During *Vařila myšička kašičku*, the mother first draws a circle in the palm of her infant and then moves successively the individual fingers of the infant’s hand, beginning with the thumb. At the same time, the mother tells a story about a mouse feeding her hungry children. The game ends when the smallest mouse (i.e., the little finger) runs to the pantry to steal some food (i.e., mother runs her fingers along the infant’s body toward the mouth or the armpit). During the game *Kovej, kovej, kováříčku* the infant becomes a horse. The game begins with the horse being studded with horseshoes. To do this, the mother gently taps with her hand on the sole of the infant’s foot. Thereafter, the horse is given different cereals, which the mother symbolically indicates by putting them in the hands of her infant.

### Infant Engagement in Social Game Routines

To find out in which situations mothers initiated social game routines, we first examined infant behaviors in the time period before the first game occurred. A Wilcoxon singed rank test showed that infants looked significantly longer away (*M* = 55.44, *SD* = 34.31) than at the mother (*M* = 34.53, *SD* = 30.74) in the period leading up to the first game, *z* = 2.035, *p* = 0.042. There were no significant differences in infant affect displays. In addition, we combined infant social engagement behaviors 2 s before each game into three groups: engagement, disengagement, and ambivalent engagement. Engagement included gazes at mother or the combination of gazes at mother and positive affect. Disengagement was composed of gazes away from the mother or the combination of gazes away from the mother and negative affect. Ambivalent engagement included gazes at mother in combination with negative affect or gazes away from the mother in combination with positive affect. The frequencies of these combined behavioral categories were adjusted to the frequency of games for each dyad. The Friedman test showed significant differences among the repeated measures, χ^2^ = 19.183, *p* < 0.001. Wilcoxon’s pairwise comparisons showed that mothers initiated games significantly more often when infants were disengaged (*M* = 58%, *S.E.* = 6.77) as compared to when they were already engaged with each other (*M* = 33.98%, *S.E.* = 5.37), *z* = − 1.959, *p* = 0.050, or when their engagement was ambivalent (*M* = 7.82%, *S.E*. = 6.77), *z* = −3.645, *p* < 0.000. Moreover, compared to ambivalent engagement, mothers initiated games significantly more often when they were already engaged with their infants, *z* = −3.905, *p* < 0.000.

**FIGURE 2:**
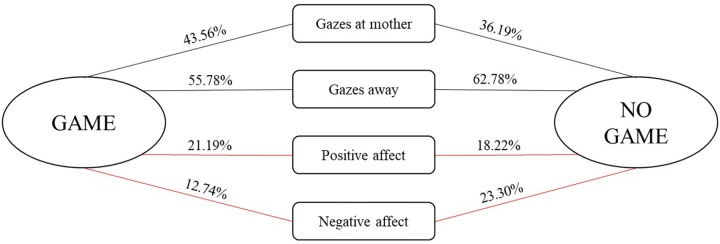
Conditional probabilities (expressed in %) for the occurrence of infant social behaviors depending on whether they were followed by a game routine or another form of social interaction. Red lines indicate (marginally) significant pairwise comparisons between conditional probabilities for the occurrence of a particular behavior being followed by a game and another form of social interaction.

We further examined the conditional probabilities for the occurrence of infant social engagement behaviors depending on whether they were followed by a game routine or another form of social interaction (see **Figure 2**). Wilcoxon’s pairwise comparisons showed that infants displayed more positive affect, *z* = −1.884, *p* = 0.06, and less negative affect, *z* = −2.935, *p* = 0.003, during game routines in comparison to the rest of the interaction.

Finally, we compared infant social engagement before and after game routines to examine whether games were instrumental in changing infants’ behaviors. Friedman’s ANOVA revealed no significant changes in infants’ behaviors, suggesting that social game routines have no sustainable effect on infants’ engagement.

### Relationship Between Social Game Routines and OT

There was a substantial amount of missing OT data for both mothers (OT1 = 25.6%; OT2 = 14%; OT3 = 18.6%; OT4 = 32.6%) and infants (OT1 = 39.5%; OT2 = 30.2%; OT3 = 34.9%; OT4 = 48.8%) that was due to either an insufficient volume of saliva or an error in computing the OT curve. Little’s MCR test showed that OT data were missing completely at random (*p* = 0.540). Moreover, we found no differences between dyads with and without missing data on any of the background or the main behavioral variables. Consequently, the multiple imputation method was used to replace missing OT values. All subsequent analyses were computed with original data as well as imputed data (pooled results), and results with both data sets are reported.

We employed three different ways of measuring OT (see also **Figure 1**). First, we calculated individual OT values for each of the four time points. Second, we calculated the area under the curve (AUC) of all four OT measurements with respect to ground (AUC_G_). AUC_G_ gives information about the total hormonal output, and takes into account differences between single measurements from each other and the distance of these measurements from the ground. This approach is frequently used to comprise information contained in repeated measurements (Pruessner et al., 2003). Third, in order to assess the individual changes in OT, we computed the area under the curve with respect to increase (AUC_I_), for the time interval before (OT2) and after (OT3) the natural interaction. The AUC_I_ is a measure of AUC with reference to the first value. It ignores the distance from zero and, thereby, emphasizes the sensitivity of the system and changes over time (Pruessner et al., 2003). The AUC_I_ allows comparing individual changes in reactivity, rather than simply comparing means over time and, therefore, represents a more advantageous approach to measuring interpersonal differences. Negative values indicate a decrease and positive values an increase in OT. Descriptive statistics for original and imputed data of all OT values are reported in **Table 4**, and bivariate correlations of all OT measures within and between mothers and infants are provided in **Table 5**. Maternal and infant AUC_G_ were significantly positively correlated [original data: *r*(24) = 0.450, *p* = 0.027; imputed data: *r*(43) = 0.433, *p* = 0.004], while their AUC_I_ were significantly negatively correlated [original data: *r*(21) = −0.440, *p* = 0.046; imputed data: *r*(43) = −0.553, *p* < 0.001].

**Table 4:** Descriptive statistics for original and imputed OT measures (in pg/ml) of the present study.

		*M*	*SD*	Range
**Maternal OT**				
OT1	Original (*n* = 32)	168.6	112.2	29.43–463.2
	Imputed (*N* = 43)	170.7	99.11	
OT2	Original (*n* = 37)	166	102.3	17.39–387.4
	Imputed (*N* = 43)	171.5	96.39	
OT3	Original (*n* = 35)	170.6	102.8	12.77–410.4
	Imputed (*N* = 43)	171.9	93.06	
OT4	Original (*n* = 29)	162.5	108	36.7–440.2
	Imputed (*N* = 43)	157	93.62	
AUC_G_	Original (*n* = 36)	434.15	295.79	100.24–1120.54
	Imputed (*N* = 43)	336.15	170.56	
				47.71–723.26
AUC_I_	Original (*n* = 34)	4.27	30.00	−75.58–66.62
	Imputed (*N* = 43)	0.22	30.29	−80.46–66.62
**Infant OT**				
OT1	Original (*n* = 26)	169.5	132.6	35.13–503
	Imputed (*N* = 43)	185	109.6	
OT2	Original (*n* = 30)	193.9	119.3	29.72–485.1
	Imputed (*N* = 43)	202.4	104.5	
OT3	Original (*n* = 28)	182.1	116.8	11.5–441
	Imputed (*N* = 43)	177.6	100.4	
OT4	Original (*n* = 22)	159.5	76.09	52.04–320
	Imputed (*N* = 43)	161	61.76	
AUC_G_	Original (*n* = 26)	447.67	291.42	123.43–1277.42
	Imputed (*N* = 43)	359.36	158.79	
				94.71–808.60
AUC_I_	Original (*n* = 24)	−7.79	54.19	−163.59–63.32
	Imputed (*N* = 43)	−12.40	49.68	−163.59–80.72

*AUC_G_, the area under the curve with respect to ground. AUC_I_, the area under the curve with respect to increase.*

**Table 5A:** Bivariate relations between maternal and infant baseline OT at the four time points (original data).

	mOT1	mOT2	mOT3	mOT4	iOT1	iOT2	iOT3	iOT4
mOT1	–							
mOT2	0.823^∗∗∗^	–						
mOT3	0.817^∗∗∗^	0.829^∗∗∗^	–					
mOT4	0.648^∗∗∗^	0.648^∗∗∗^	0.735^∗∗∗^	–				
iOT1	0.472^∗^	0.411^∗^	0.551^∗∗^	0.332	–			
iOT2	0.433^∗^	0.297	0.517^∗∗^	0.596^∗∗^	0.574^∗∗^	–		
iOT3	0.446^∗^	0.352	0.253	0.248	0.390^+^	0.615^∗∗∗^	–	
iOT4	0.478^∗^	0.431^+^	0.538^∗^	0.339	0.678^∗∗^	0.679^∗∗∗^	0.645^∗∗^	–

*^+^p < 0.06, ^∗^p < 0.05, ^∗∗^p < 0.01, ^∗∗∗^p < 0.001. i, infant; m, mother.*

**Table 5B:** Bivariate relations between maternal and infant baseline OT at the four time points (imputed data).

	mOT1	mOT2	mOT3	mOT4	iOT1	iOT2	iOT3	iOT4
mOT1	–							
mOT2	0.826^∗∗∗^	–						
mOT3	0.816^∗∗∗^	0.796^∗∗∗^	–					
mOT4	0.570^∗∗∗^	0.559^∗∗∗^	0.718^∗∗∗^	–				
iOT1	0.333^∗^	0.249	0.427^∗∗^	0.117	–			
iOT2	0.314^∗^	0.245	0.490^∗∗∗^	0.563^∗∗^	0.471^∗∗∗^	–		
iOT3	0.384^∗^	0.332^∗^	0.224	0.246	0.395^∗∗^	0.530^∗∗∗^	–	
iOT4	0.447^∗∗^	0.403^∗∗^	0.510^∗∗∗^	0.336^∗^	0.514^∗∗∗^	0.548^∗∗∗^	0.664^∗∗∗^	–

*^∗^p < 0.05, ^∗∗^p < 0.01, ^∗∗∗^p < 0.001. i, infant; m, mother.*

Spearman correlational analyses between the game variables and OT variables showed that, for original data, game rate was significantly negatively correlated with infant OT3, *r*(28) = −0.413, *p* = 0.029, and infant AUC_I_, *r*(24) = −0.425, *p* = 0.038, as well as positively with maternal AUC_I_, *r*(34) = 0.531, *p* = 0.001. Similarly, for imputed data, game rate was marginally negatively correlated with infant OT 3, *r*(43) = −0.296, *p* = 0.054, and positively with maternal AUC_I_, *r*(43) = 0.376, *p* = 0.013.

Moreover, for original data, time spent playing games during the interaction was negatively related to infant OT3, *r*(28) = −0.405, *p* = 0.032, and positively to maternal AUC_I_, *r*(34) = 0.368, *p* = 0.032. For imputed data, there was also a trend for a relationships between time spent playing games during the interaction and maternal AUC_I_, *r*(43) = 0.279, *p* = 0.070. No other correlations reached significance.

## Discussion

Results of the present study suggest that social game routines are an inherent part of early mother–infant interactions. In this sample, almost 77% of mother–infant dyads spontaneously engaged in game routines during their interactions without being instructed to do so. Thus far, no research has examined naturally occurring game routines during early mother–infant interaction, and therefore the present results cannot be compared with existing evidence. However, the large prevalence as well as variety of game routines found in the present study may be attributable to the particulars of the examined sample. Specifically, the sample examined in the present study consisted of Czech women with their infants, and the Czech language has a particularly large repertoire of social game routines that are well-known in the general public. Moreover, playing mothers reported, on average, higher years of education than non-playing mothers, suggesting that engaging in structured game routines with infants may depend on maternal educational status. Thus, it remains an open question, whether these results are representative of other linguistic, societal and/or cultural backgrounds.

Despite these limitations the present findings show that social game routines occurred naturally during interactions between mothers and their 4-month-old infants, and were clearly distinguishable from the remaining activities during the interaction. A major problem of play research remains the difficulty in defining what constitutes a play activity with the consequence that any social activity cannot be clearly differentiated from play. In an attempt to identify play in various species, including humans, Burghardt (2011) has proposed five criteria of play. Accordingly, playful behavior is (1) not necessary for current survival; (2) spontaneous, voluntary, intentional, pleasurable, rewarding, reinforcing, or autotelic; (3) not fully functional, because it incorporates incomplete, exaggerated, awkward, or precocious elements, or involves modified or sequenced behavior patterns; (4) being repeated in a similar form; and (5) initiated in a “relaxed field,” when all basic needs are provided for. All of these criteria can be applied equally well to social/interactive as well as playful activities. The lack of a good operational definition of play makes it almost impossible to systematically examine early playful activities. Interestingly, we instantly recognize play when we see it, and the present study shows that focusing on particularly structured playful behavior (i.e., game routines) could provide a way to circumvent the definitional challenges. Of course, the research assistants coding the data for the present study were Czech, and, as argued above, the knowledge of game routines is widely spread in the general Czech population. Thus, it is unclear whether coders who do not speak Czech could identify game routines in this study. Yet, we have provided some reference points for a possible operational definition of game routines, and it remains for future research to ascertain its applicability across different samples.

Next, we were interested in the context in which mothers initiated social game routines during the interaction. Our findings suggest that mothers most often began playing game routines when infants were not engaged with them, even more so than when there was an ongoing engagement between mothers and infants. It seems that mothers used game routines as a strategy to regain their infants’ attention or interest in the interaction. This could have been a function of the observation situation – mothers were asked to interact with their infants as they would at home in a strange environment, with cameras directed at them, with the pressure to ‘perform’ and, most importantly, without the possibility to use any objects (e.g., toys). In such a possibly stressful context, engaging in a structured playful routine with their infants may have provided a way for mothers to create a comfortable zone for themselves as well as redirect the infant’s attention back to them when the interaction went astray. Again, it remains to be examined how social game routines are used in other contexts, for example during interactions at home.

Confirming our hypothesis, the probability was significantly higher for infants to display positive affect during game routines than during other social activities, while the probability for negative affect during game routines was significantly lower than during the rest of the interaction. Thus, playing games may have fulfilled one of the main goals of a playful interaction – creating enjoyment (Stern, 1974; Ratner and Bruner, 1978; Papoušek and Papoušek, 1995), which corroborates research examining older children (Crawley et al., 1978; Ross and Kay, 1980). Fantasia et al. (2014b) also showed a general tendency toward positive affectivity during game routines in 3-month-old infants as compared to modified games. Thus, it is possible that infant positive affect during a game is a result of their recognition and understanding of the familiar structure of the ongoing game routine (Ratner and Bruner, 1978; Fantasia et al., 2014b). In fact, our finding that there were no changes in infant behaviors from before to after playing game routines with their mothers would support such an argument. That is, while the probability for positive affect during social game routines is higher than for other types of social activities, playing games does not seem to have a sustainable impact on the infants’ mood. In line with the argument presented above, playing social game routines may create a comfort zone in an otherwise stressful social environment, which then elicits positive affect in infants that serves as a feedback loop back to the mother to signal to her that the interaction is enjoyable. This interpretation could also explain the large numbers of social game activities found.

Interestingly, the results pertaining to the third goal of the present study seem to support such theorizing. Specifically, we found that the number of game routines played and the time spent playing them during the interaction was positively related to maternal increase in OT from before to after the interaction. Because the current data is correlational in nature, any implications of directionality remain speculative. Nevertheless, findings of the present study are consistent with current literature on the role of OT in early social interactions, and particularly its associations with interactional synchrony (e.g., Feldman et al., 2010a, 2011; Gordon et al., 2010b,c). The concept of synchrony puts focus on time as a central parameter and is characterized by ‘co-occurrence’ or ‘match’ between the infant’s and parental behaviors (Feldman, 2006, 2007). This research has consistently shown that synchrony between parental and infant interactive behaviors is linked to both parental and infant OT (e.g., Feldman et al., 2010a, 2011; Gordon et al., 2010b,c). In the present study, mothers who took more initiative to play structured game routines during the interactions with their infants may have felt more comfortable during the laboratory observation, especially when receiving positive affect from their infants in feedback, and this was reflected in an increase in maternal OT from before to after the interaction. This argument would also be consistent with the fact that mothers were more compelled to initiate game routines with infants when they were disengaged from them in the interaction. Thus, the motivation for playing structured game routines during laboratory parent–infant interactions may be to connect, coordinate and thus establish behavioral patterns and bonds between parents and infants as an active strategy to bring the interaction to a new level, rather than simply for the sake of playing.

Additionally, it could be hypothesized that playing social game routines activates maternal caretaking behaviors that are associated with increased levels of OT and prolactin (see also Panksepp, 1998). This hypothesis further suggests that caregiving quality may play a role in the usage of game routines, particularly as a strategy in possibly stressful situations. On the one hand, previous research has shown that the degree of synchrony between parents and infants moderated the relation between parental and infant levels of salivary OT: under conditions of high synchrony, infants whose parents had high OT levels had also significantly higher OT levels compared to infants whose parents had relatively low OT levels (Feldman et al., 2010b). In contrast, no differences were observed in infant OT levels when interactional synchrony of the dyad was low (Feldman et al., 2010b). On the other hand, there is evidence that both play and OT have stress-reducing qualities (Watamura et al., 2003; Marazziti et al., 2006; Numan and Woodside, 2010; Guzman et al., 2013; Elmadih et al., 2014). Specifically, OT released during interpersonal stress modulates the psychological reactivity to these experiences, inducing calmness and increasing motivation for social interactions (Uvnäs-Moberg, 1998). Interestingly, Elmadih et al. (2014) suggested that OT is released to reduce interpersonal stress and anxiety particularly when maternal sensitivity is low. Correspondingly, it is possible that mothers used game routines to relieve the stress arising from the observation situation, which could have resulted in an increase in their salivary OT, and, in turn, heightened their caretaking behaviors. Recent observations of parental use of mobile devices during their interactions with children showing the adverse effects of distracted parenting would support this conclusion. That is, distracted parents showed an overall reduction in their active engagement (e.g., Kirkorian et al., 2009), slow or absent responsiveness, as well as reduced sensitivity (see Kildare and Middlemiss, 2017, for review). Naturally, such interferences of caretaking behaviors would not only increase the overall stressful experience, but also prevent playing social game routines with the aim to reconnect with the infant. Further research needs to take into consideration the factors and circumstances that may facilitate or impede the occurrence of social game routines.

Panksepp (1998) proposes what he calls the PLAY system that is located in the subcortices, particularly in brain regions rich in opioids and dopamine, and there is now substantial evidence showing that opioids can increase playful behaviors (see Panksepp, 1998, for review). In contrast, OT was found to suppress play behavior in juvenile rats (Panksepp, 1998), which seems at odds with the above-discussed findings of an increase in maternal OT after interactions rich in social game routines. However, results of the present study also showed that particularly playful mother–infant interactions (i.e., more games and more time spent playing game routines) were associated with less infant OT sampled after the interaction (i.e., OT 3) as well as a decrease in infant OT from before to after the interaction. There seems to be an interesting interaction between play and social interaction at the neurochemical level: while opioids can increase play, they simultaneously can decrease the desire for social interaction (Panksepp, 1998). Thus, if there is play, then there is no motivation for social interaction, and vice versa, which could explain the inverse relationship between playing games and infant OT in the present study. Because dyads in the present study played a lot, this may have affected infant OT levels, which is particularly evident in the OT sampled after the natural interaction. Relatedly, it is also possible that the decrease in infant OT from before to after playful interactions reflects a stress-regulating mechanism of the dyad. That is, if mothers and infants experienced the observation situation in the laboratory as particularly stressful, then mothers may have initiated game routines as an attempt to coordinate behaviors with their infant. While in mothers this strategy could have heightened their caretaking behaviors and thus increased their OT level (see discussion above), in infants it could be responsible for a reduction in stress and a corresponding decrease in OT. Thus, maternal caretaking efforts via playing may have moderated infants’ stress response.

The present study’s specifications may limit the conclusions presented here. First, the study took place in a laboratory setting, which, as discussed above, may explain the high numbers of game routines observed. Maternal behavior toward their infants is affected by a laboratory context (Belsky, 1980), and although care was taken for mothers and their infants to feel comfortable, we cannot rule out maternal performance-anxiety, which would not only affect their behaviors, but also their OT levels (e.g., Marazziti et al., 2006). Second, the linguistic and cultural background of the examined sample strongly limits the possibility to generalize the found results to other populations. As already suggested, the Czech language has a rich repertoire of social game routines that are widely known. The occurrence of social game routines during natural interactions thus remains to be examined in future research to corroborate the game rates found in the present study. Third, the sampling of salivary OT may have affected the present findings, because the coordination mechanism between OT release in the central and peripheral nervous system is not fully understood. Moreover, unlike previous studies using saliva samples (e.g., Carter et al., 2007; Feldman et al., 2010a,b, 2011), we analyzed the samples directly without extracting and concentrating the samples. This might have caused the missing OT data and elevated absolute OT values. Thus, further research validating ELISA analysis of OT in saliva is critically required (e.g., Bosch, 2014). Other potentially limiting factors include recruitment strategies, which resulted in a sample representing middle to upper class, highly educated families and over-recruitment of first-time mothers, the correlational nature and a small sample size. Despite these limitations, the present results provide first compelling evidence of naturally occurring social game routines and the different role of the maternal and infant OT system during playful interactions.

Animal research has shown that play is deeply embedded in the mammalian brain (Panksepp, 1998), and, similarly, it is argued to be essential in the core biological functioning in humans (Kestly, 2014). Thus, there may be an evolutionary advantage for human infants to engage in play. During play, infants can encounter and experiment with different emotions, thoughts, roles and rules, which enables them to have particular expectations about their exchanges with others and thus be intrinsically cooperative. Mainstream views on cooperation see cooperative actions as a result of infants’ ability to infer others’ thoughts and plans, and combine them to build their co-actions in some shared way (e.g., Tomasello, 2009). This view presupposes high-level mentalizing abilities, which very young infants do not possess. An alternative view sees cooperation as a property of interaction processes, not as an individual attitude toward another person (Fantasia et al., 2014a). Accordingly, no high-level mental abilities are needed for cooperation to take place. Recent evidence supports this view by showing that very early in life infants form expectations about and adjust to others’ actions during daily routines (e.g., Nomikou and Rohlfing, 2011; Rączaszek-Leonardi et al., 2013; Reddy et al., 2013). Playing structured social games may be considered another form of such joint routines that helps infants to assume different roles and experience variations of social exchanges (e.g., Fantasia et al., 2014b), and consequently become skilled cooperative agents as they participate in them (Rączaszek-Leonardi et al., 2013). Thus, early social games may support the development of complex social competencies, because they enable infants to become increasingly skilled in their social participation without the need for higher-level mentalizing.

## Ethics Statement

This study was carried out in accordance with the recommendations of the Ethics Committee of the Institute of Psychology, Czech Academy of Sciences with written informed consent from all subjects. All subjects gave written informed consent in accordance with the Declaration of Helsinki. The protocol was approved by the Ethics Committee of the Institute of Psychology, Czech Academy of Sciences.

## Author Contributions

GM has made a substantial contribution to the study conception, design, formulation of the theoretical arguments, data analyses, and interpretation of data.

## Conflict of Interest Statement

The author declares that the research was conducted in the absence of any commercial or financial relationships that could be construed as a potential conflict of interest.
