# Supplementary material for: The Games Infants Play: Social Games During Early Mother–Infant Interactions and Their Relationship With Oxytocin
Source: Front Psychol. 2018 Jun 25;9:1041. doi: 10.3389/fpsyg.2018.01041 (PMC6026809; doi:10.3389/fpsyg.2018.01041)
Supplement: Supplementary file 1 [file Table_1.PDF]

Table S1. List of all Game Routines Observed in the Present Study Including Their Occurrence Frequency.

**Occurrence frequency: 10+**
Paci, paci, pacičky (17)
Varila myšička (13)
Occurrence frequency: 5-9
Kovej kovaříčku (9)
Leze po železe (6)
Brambora (5)
Berany duc (5)
**Occurrence frequency: 2-4**
Běžela myška (4)
Žába leze (3)
Šiju boty (3)
To je máma (2)
Ťapi ťapičky (2)
Skákal pes (2)
Prší prší (2)
Houpi (2)
Holka modrooká (2)
Hlava ramená (2)
Dešťové kapky (2)
**Occurrence frequency: 1**
Všechny tvoje prstičky
Vločka

Šel pán doktor

Spadla šiška

Spadla muška

Sluníčko

Sedí papír

Pravá noha

Pec nám spadla

Pásla ovečky

Nikoho to nenapadlo

Matlafousek

Mámo, táto, v komoře je myš

Mám dvě ouška

Levá pravá

Vrána letí

Křeček

Kdo se vleče

Kapaly kapky

Jeden, dva, tri, štyri

Jede z kopečka

Ja mám koně

Já jsem z Kutný hory

Hopsasa

Hopsa Hejsa

Honza jede

Čižíček

Bumtarata

Běží liška
